# Activation of the human immune system by chemotherapeutic or targeted agents combined with the oncolytic parvovirus H-1

**DOI:** 10.1186/1471-2407-11-464

**Published:** 2011-10-26

**Authors:** Markus Moehler, Maike Sieben, Susanne Roth, Franziska Springsguth, Barbara Leuchs, Maja Zeidler, Christiane Dinsart, Jean Rommelaere, Peter R Galle

**Affiliations:** 1 First Department of Internal Medicine, University Medical Center of the Johannes Gutenberg University of Mainz, 55131 Mainz, Germany; 2 Infection and Cancer Program, Department F010, Deutsches Krebsforschungszentrum Heidelberg, 69120 Heidelberg, Germany; 3 Institut National de la Santé et de la Recherche Médicale Unité 701, Deutsches Krebsforschungszentrum Heidelberg, 69120 Heidelberg, Germany

## Abstract

**Background:**

Parvovirus H-1 (H-1PV) infects and lyses human tumor cells including melanoma, hepatoma, gastric, colorectal, cervix and pancreatic cancers. We assessed whether the beneficial effects of chemotherapeutic agents or targeted agents could be combined with the oncolytic and immunostimmulatory properties of H-1PV.

**Methods:**

Using human *ex vivo *models we evaluated the biological and immunological effects of H-1PV-induced tumor cell lysis alone or in combination with chemotherapeutic or targeted agents in human melanoma cells +/- characterized human cytotoxic T-cells (CTL) and HLA-A2-restricted dendritic cells (DC).

**Results:**

H-1PV-infected MZ7-Mel cells showed a clear reduction in cell viability of >50%, which appeared to occur primarily through apoptosis. This correlated with viral NS1 expression levels and was enhanced by combination with chemotherapeutic agents or sunitinib. Tumor cell preparations were phagocytosed by DC whose maturation was measured according to the treatment administered. Immature DC incubated with H-1PV-induced MZ7-Mel lysates significantly increased DC maturation compared with non-infected or necrotic MZ7-Mel cells. Tumor necrosis factor-α and interleukin-6 release was clearly increased by DC incubated with H-1PV-induced SK29-Mel tumor cell lysates (TCL) and was also high with DC-CTL co-cultures incubated with H-1PV-induced TCL. Similarly, DC co-cultures with TCL incubated with H-1PV combined with cytotoxic agents or sunitinib enhanced DC maturation to a greater extent than cytotoxic agents or sunitinib alone. Again, these combinations increased pro-inflammatory responses in DC-CTL co-cultures compared with chemotherapy or sunitinib alone.

**Conclusions:**

In our human models, chemotherapeutic or targeted agents did not only interfere with the pronounced immunomodulatory properties of H-1PV, but also reinforced drug-induced tumor cell killing. H-1PV combined with cisplatin, vincristine or sunitinib induced effective immunostimulation via a pronounced DC maturation, better cytokine release and cytotoxic T-cell activation compared with agents alone. Thus, the clinical assessment of H-1PV oncolytic tumor therapy not only alone but also in combination strategies is warranted.

## Background

The inherent host tumor immunosurveillance system combats the formation and growth of tumors, mainly relying on the interaction of effector immune cells with the tumor cells [1]. Activation of tumor-specific cytotoxic T-lymphocytes (CTL) requires presentation of tumor-associated antigens (TAAs) primarily by dendritic cells (DC), in addition to the helper functions of CD4^+ ^cells [2]. For this reaction to proceed, immature DC with high endocytic activity must differentiate into mature DC with increased expression of co-stimulatory molecules that prime and boost T-cell and B-cell functions [3]. The implementation of these immune reactions into anti-tumor therapy is desirable but cannot be satisfactorily achieved in many situations through classical systemic therapy alone.

Recently, we and other groups demonstrated the induction of increasing immune reactions using oncolytic viruses in both syngeneic mouse and human *ex vivo *and *in vivo *tumor xenograft models [4-6]. Parvoviruses or other viruses employed as therapeutic gene vectors are used to stimulate the immune system [6-8]. However, many of these vectors are restricted by their pathogenicity and adverse immunological side-effects [9]. Non-pathogenic parvovirus H-1 (H-1PV), depending on the target cells and culture conditions, induced apoptosis or autophagy-like cell death [8,10-12]. Besides genuine oncolytic activity, Bhat et al showed that targeted tumor cell H-1PV infection and the improved recognition as target cells by natural killer (NK) cells leads to an amplification of NK cell-mediated immune response [13]. Furthermore, H-1PV efficiently induced viral oncolysis in Burkitt's lymphoma cells, including those resistant to apoptosis induction by rituximab [14]. In addition, H-1PV could activate human anti-tumor immune response by adoptive transfer and an abortive H-1PV infection of human peripheral blood mononuclear cells (PBMC) [15]. Thus, H-1PV efficiently activated the human immune system and may potentially support classical systemic chemotherapy and/or new molecular targeted agents in the treatment of human cancer patients [10,16].

Parvoviruses are small nuclear DNA viruses that replicate during S-phase of the cell cycle. H-1PV efficiently infects human tumor cells, including melanoma, hepatoma, colon and gastric cancer cells [8,10,17]. Moreover, parvovirus productive lytic infection resulted in reduced incidence of spontaneous, virally, and chemically induced tumors in animals [8,18]. In contrast to these fast-replicating cells, human immune cells and primary hepatocytes cannot be infected or lysed [8,10,15]. Moreover, recombinant parvoviruses that are deficient in replication have been engineered to deliver immunostimulating molecules to increase their anti-tumor properties [8,18,19]. We further reported that immunogenic heat shock proteins are released during the process of H-1PV-induced killing of human melanoma cells,[6,10] and demonstrated increased phagocytosis of H-1PV-induced tumor cell lysates (TCL) leading to increased maturation of DC. These activated DC improved tumor antigen presentation with stimulation of TAA-specific CTL via cross presentation [6].

So far, the immunological effects of combining H-1PV and conventional chemotherapeutic agents or newer targeted agents are yet unknown. Thus, the aim of the current study was to analyze the putative synergistic biological and immunological effects of H-1PV combined with cisplatin, vincristine or the multi-tyrosine kinase (TK) inhibitor, sunitinib, in human tumor and immune cells. We employed human melanoma models, which allowed the study of immune responses in the context of corresponding HLA-restricted human DC. This human *ex vivo *tumor model with tumor-specific autologous CTL clones was also used with HLA-A2-positive and HLA-A2-negative melanoma variant cells [6]. Since new molecular targeted therapies, including sunitinib, erlotinib or sorafenib, are increasingly being approved for treatment of many human cancers, due to their combined tumor-suppressive and anti-angiogenic effects,[20,21] their combinations with H-1PV may be even more attractive to induce CTL. Therefore, we aimed to develop a more effective DC-mediated immune stimulation by combining chemotherapeutic or targeted agents with H-1PV.

## Methods

### Human cancer cells and primary human immune cells

Human melanoma cell lines MZ7-Mel, SK29-Mel-1 and SK29-Mel-1.22 were cultured as previously described [10]. The autologous melanoma line MZ7-MEL was established from a splenic metastasis in 1988. MZ7-MEL expressed MAGE-A3, tyrosinase, Melan-A/MART-1 and gp100 [22]. The SK29-Mel-1.22 cell line is a selected HLA-A2 negative (A2^-^) variant of HLA-A2-positive (A2^+^) SK29-Mel-1 cells [23]. The HLA-A2-restricted CTL line, CTL IVSB, directed against the TAA tyrosinase peptide 369-376 [24] was derived from an autologous mixed lymphoid tumor cell culture of the SK29-model. These cells and the EBV-transformed B-cell line (MZ-EBV-B) were obtained as a gift from T. Woelfel (Mainz, Germany). CTL were maintained in long-term culture by passaging every 4-7 days [24]. Primary human immune cells (monocytes, immature and mature DC) were derived from buffy coats of healthy blood donors in the Department of Transfusion Medicine of Johannes Gutenberg University Mainz (Mainz, Germany) and used as described [6,25]. For the use of samples from healthy blood donors no ethical approval was needed.

### Virus preparation and infection of tumor cells

Wild-type H-1PV was produced in NB-324K cells and purified on iodixanol gradients. For wild-type virues titers are determined by plaque assays as previously described [26] and the multiplicity of infection (MOI) is given by the number of plaque-forming units (PFU). To infect tumor cells with H-1PV, medium of exponentially growing cell cultures were removed and then incubated for one hour with H-1PV at a MOI of 20 PFU/cell in minimal amount of complete medium (Minimum Essential Medium, 5% FCS, 1% glutamine, 1% penicillin/streptomycin; Life Technologies, Karlsruhe, Germany) and than fill up to appropriate amount of medium according to the size of dishes, plates and flasks. Cells were cultured for up to 10 days post infection (p.i.).

### Cell treatment

For combined treatment, cells were firstly infected with H-1PV in complete medium (described above) and one hour or 24 hours after infection, chemotherapeutic agents or sunitinib were added by adding appropriate amount of medium and cells were incubated until analysis. Cisplatin and vincristine were purchased from Gry Pharma GmbH (Kirchzarten, Germany) and freshly dissolved in medium to a concentration from 0.1 μg/ml to 5 μg/ml, a concentration of 0.1 μg/ml was used for analysis. Sunitinib (sunitinib malate, SUTENT^®^; Pfizer Inc.) was provided by Pfizer (Pfizer, NY, USA), and was dissolved in dimethylsulfoxide (Invitrogen, Karlsruhe, Germany) from 1-5 μg/ml and a concentration of 5 μg/ml was used for analysis.

### Virus-driven transgene expression and protein analyses

For measurement of transient H-1PV expression, virus particles were quantified by luciferase expression using the NS-proficient recombinant parvovirus hH1Δ1600luc (carries firefly luciferase gene instead of viral caspid proteins) as described previously [8]. Infections were performed for 1 hour at a MOI of 10-2 RU/cell. For recombinant viruses titers are determined by infected cell hybridization assays and are expressed as replication units (RU) per milliliter of virus suspension. Finally, cells were harvested on days 3 and 4 p.i. Luciferase activities were determined with a Luminometer (Berthold, bad Wildbad, Germany) and expressed as level of induction relative to control. NS1 was analyzed by western blotting as previously described [8,11]. Blots were incubated with the rabbit polyclonal antibody SP8 (1:1000) directed against carboxy-terminal peptides of NS1,[27] and processed for enhanced chemoluminescence detection (Amersham Pharmacia Biotech, Freiburg, Germany).

### H-1PV-induced cytotoxicity and analysis of apoptosis

To quantify cellular cytotoxicity, cells infected with H-1PV and/or treated with chemotherapeutic agents or sunitinib were grown for up to 6 days and stained with crystal violet for 1 hour. Measurements were performed at 550 nm at day 4 and 6 p.i. The growth inhibition (percentage of survival) was defined as percentage reduction of photometric absorption measurements of H-1PV-infected versus non-infected cell cultures. The absorption was measured via an enzyme-linked immunosorbant assay (ELISA) reader. The results were presented as relative to the control value (untreated cells). Cell viability of H-1PV infection 1 or 24 hours p.i., in addition to sunitinib treatment alone or in combination with H-1PV, was monitored by the 2-(4,5-dimethyltriazol-2-yl)-2,5-diphenyl tetrazolium bromide (MTT, Biomol, Hamburg, Germany) colorimetric assay. The absorbance was measured at 570 nm. Percent viability was defined as the relative absorbance of treated versus untreated control cells. To quantify the percentage of apoptotic cells in H-1PV-infected cultures (MOI = 20 PFU/cell), adherent cells were dissociated via trypsinization and collected 3 days p.i. by centrifugation at 800 × g. The harvested cells were processed as described previously [11]. Levels of apoptosis were assessed by FACScan flow cytometry with Cell Quest software (Becton Dickinson, Heidelberg, Germany) according to the manufacturer's instructions [8].

### Immunologic analysis for DC phagocytic activity, maturation, cross presentation and cytokine release

For phagocytic activity and maturation, DC were labeled with PKH2 and melanoma cells with PKH26. Labeled melanoma cells were infected with H-1PV (MOI = 20 PFU/cell). On day 10 p.i., TCL from H-1PV-infected melanoma were incubated for 2 days with PKH2-stained immature DC at a ratio of 1:3 in a 24-well plate. Non-infected melanoma cells, UV-irradiated (200 J/m^2^) and ultrasonicated tumor cells were stained with PKH26 prior to UV-irradiation and sonication and used as controls. To gate out mature DC from immune and dead cells, cells were treated as described previously [6]. After 1 day of co-culture, PKH2-labeled DC were analyzed for uptake of PKH26-stained melanoma TCL by FACS. DC staining was performed with phycoerythrin-labeled antibodies against human CD80, CD83 and CD86, and controlled with appropriate isotype-matched antibodies as previously described [6]. Expression levels were measured by FACScan after immature DC were incubated with untreated melanoma cells, UV-irradiated melanoma cells or H-1PV-infected melanoma cells 10 days p.i..

To explore the maturation status of DC incubated with H-1PV-infected TCL combined with chemotherapeutic agents, SK29-Mel cells were infected with H-1PV (MOI = 20 PFU/cell). After one hour of viral infection, either vincristine or cisplatin was added into the medium. These 6-days differently incubated melanoma cells were co-cultured with immature DC for 2 days. Immature DC were marked with phycoerythrin-labeled antibodies against human CD86 and measured by FACScan [6].

To measure inflammatory cytokines from DC, immature DC were co-cultured with TCL from H-1PV-infected or with non-infected cells for 1 day in 96-well plates in a ratio of 1:3. As a control, the production of cytokines in melanoma cells, H-1PV-infected melanoma cells, and of immature and mature DC was measured. Mature DC controls were obtained by adding a cytokine cocktail as previously described [28]. Supernatants were prepared by aspirating media from the co-culture and by diluting 1:1 with fresh medium. Tumor necrosis factor (TNF)-α and interleukin (IL)-6 levels were determined by ELISA kits (BD Biosciences Pharmingen) according to the manufacturer's protocol.

## Results

### Cytotoxicity of H-1PV-infected MZ7-Mel cells, expression of H-1PV proteins and virus-driven transgenes

**Figure 1:**
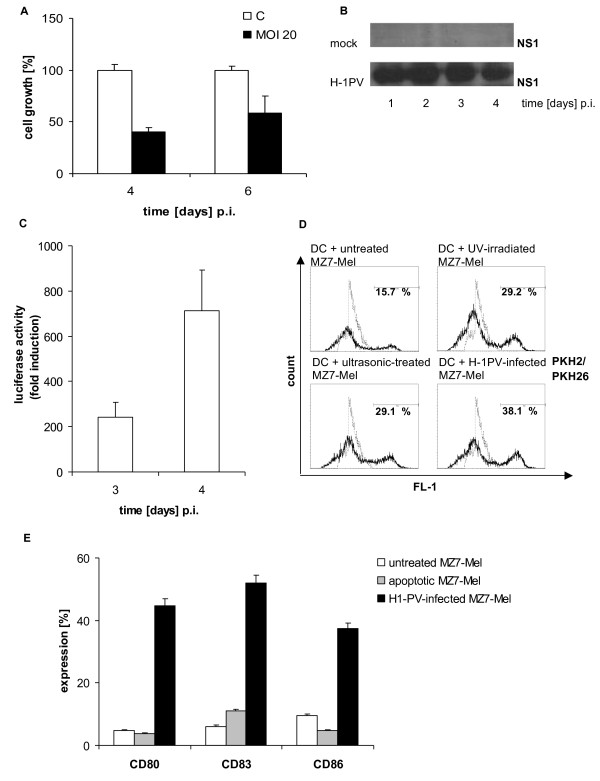
**H-1PV infection of MZ7-Mel cells**. **A: **Effect of infection on cell survival. MZ7-Mel cells were infected with H-1PV (MOI = 20 PFU/cell), grown for 4 to 6 days and stained with crystal violet. The percentage of survival is expressed as fraction of dead versus living cells by photometric analysis (mean of 24 wells, at least 2 experiments). **B: **NS1 gene expression. MZ7-Mel cells were infected and grown for 1 to 4 days. After lysis 50 μg of total proteins were equally diluted and separated on SDS-PAGE. For parvoviral protein detection, blots were incubated with the NS1-specific antibody. **C: **Virus-driven transgene expression. Cells were infected with recombinant hH1Δ1600luc (MOI = 10-2 RU/cell) and transgene activities were measured in tumor cell lysates after 3 and 4 days p.i., given in fold induction. **D: **Phagocytosis. Immature DC and MZ7-Mel cells were labeled with PKH2 and PKH26, respectively. MZ7-Mel cells were infected with H-1PV (MOI = 20 PFU/cell), cultured for 10 days, and subsequently co-cultured with iDC. After 2 days of co-culture, PKH2-labeled DC were analyzed by FACS for their uptake of PKH26-stained MZ7-Mel cell lysates. As controls, DC were incubated with melanoma cells that were either untreated, UV-irradiated (to induce apoptosis) or ultrasonic-treated (necrosis). The percentage indicates the proportion of double-positive (phagocytosing) cells. FL-1 corresponds to an area in which PKH2 and PKH26 fluorescence overlap. The dotted curve represents the untreated DC. **E: **DC maturation. The expression of CD80, CD83, and CD86 was measured by FACScan after immature DC were incubated for 2 days with untreated SK29 melanoma cells, with UV-irradiated cells, and lysates from H-1PV-infected cells (MOI = 20 PFU/cell) 10 days p.i.

The cytotoxic effect of H-1PV in MZ7-Mel cells was determined by time-dependent measurement of cell growth (Figure 1A). H-1PV infection of MZ7-Mel cells with an MOI of 20 resulted in an approximately 50% reduction of cell growth at day 4 and 6 p.i.. Six days p.i, cells continued to grow, but with a significantly reduced growth rate compared to controls. This may be due to the fact that a threshold of NS1 has to be present to induce cell cycle arrest. Until critical NS1 levels occur, cells can proliferate [29]. Time-dependent expression of the viral non-structure protein NS1 was documented in MZ7-Mel cells by western blotting at 1 day p.i. with the highest expression levels found 2 days p.i. (Figure 1B). On day 6 p.i., NS1 expression decreased, most likely due to H-1-mediated induction of apoptosis. Further results of the luciferase assay using parvovirus hH1Δ1600luc [11] indicated a more than 200-fold induction of luciferase activity 3 days p.i. compared with non-infected cells (Figure 1C). Luciferase activity was further enhanced 4 days p.i. Thus data suggest that H-1PV-induced cell killing was correlated with NS1 [8,30].

### Analysis of DC activity: phagocytosis and maturation

The immunogenicity of tumor cells was determined by phagocytosis and the presentation of tumor antigens by DC [31]. We therefore investigated the activation of DC following co-culture with H-1PV-induced MZ7-Mel lysates compared with a panel of control MZ7-Mel cell preparations. Phagocytosis of MZ7-Mel preparations by immature DC was quantified by flow cytometry (Figure 1D). The highest proportion of phagocytosing DC (PKH2 and PKH26 double-staining) was 38%, detected after co-incubation with TCLs from H-1PV-infected MZ7 Mel 10 days p.i., compared with ~29% DC coincubated with UV-irradiated and ultrasonic-treated tumor cells respectively, and 16% from untreated melanoma cells. Thus, phagocytosis in immature DC was most effectively stimulated by H-1PV-induced melanoma cell lysates.

As previously been shown [25], treatment of immature DC with a cocktail of pro-inflammatory cytokines led to DC maturation characterized by a major increase in CD80, CD83 and CD86 expression (data not shown). In order to investigate whether H-1PV-induced melanoma cell lysates modulate DC maturation, the expression of these surface markers was analyzed following incubation with H-1PV-induced melanoma cell lysates taken 10 days p.i. compared with incubation with control MZ7-Mel cell preparations (Figure 1E). DC co-cultured with melanoma cells alone did not present significant phenotypic characteristics of DC maturation (CD80: ~5%, CD83: ~6%, CD86: ~10%), and co-culture with UV-irradiated apoptotic cells led to a non-significant increase of CD83 markers only (~11%). However, DC incubated with H-1PV-induced MZ7-Mel lysates resulted in a dramatic increase of all DC maturation markers, clearly indicating DC maturation (CD80: ~45%, CD83: ~52%, CD86: ~37%) (Figure 1E).

### Cell viability after treatment with H-1PV, chemotherapeutic or targeted agents

**Figure 2:**
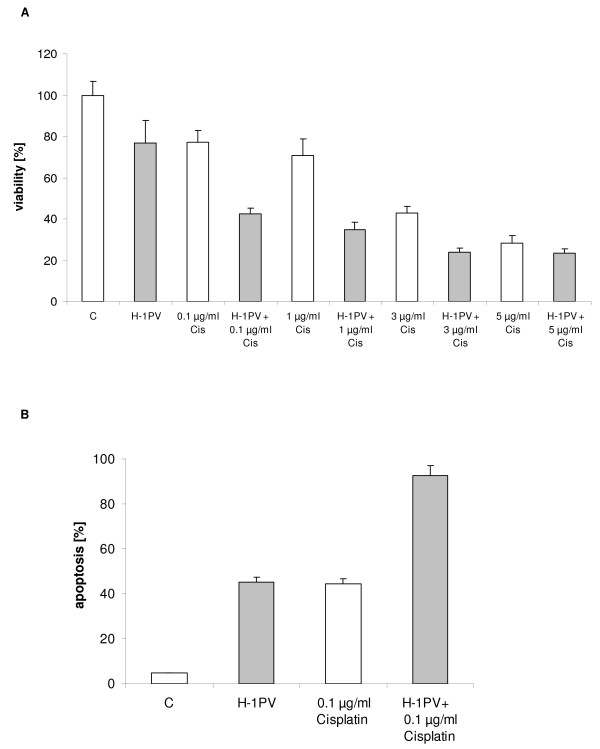
**SK29-Mel cell viability following H-1PV infection and/or treatment with chemotherapeutic agents**. **A**: Cell viability of chemotherapeutic agent-treated and/or H-1PV-infected cells (MOI = 20 PFU/cell) SK29-Mel cells was measured by MTT assay after 48 h. Absorbance was measured at 570 nm, % viability was defined as relative absorbance of treated vs untreated control cells. **B**: Apoptosis of H-1PV-and/or cisplatin-treated SK29-Mel cells 1 hour post infection (MOI = 20 PFU/cell) was measured by FACScan.

The viability of melanoma cells was assessed after exposure with cisplatin, vincristine alone, or together with H-1PV infection 24 hours p.i. using MTT assays. Cisplatin alone reduced SK29-Mel viability (Figure 2A). The reduction of cell viability was additionally enhanced when cisplatin was combined with H-1PV. Enhancement of cisplatin-mediated apoptosis was observed in the presence of H-1PV, suggesting apoptosis may contribute to the reduced viability observed with the combination (Figure 2B). Similar effects were demonstrated with vincristine (data not shown).

**Figure 3:**
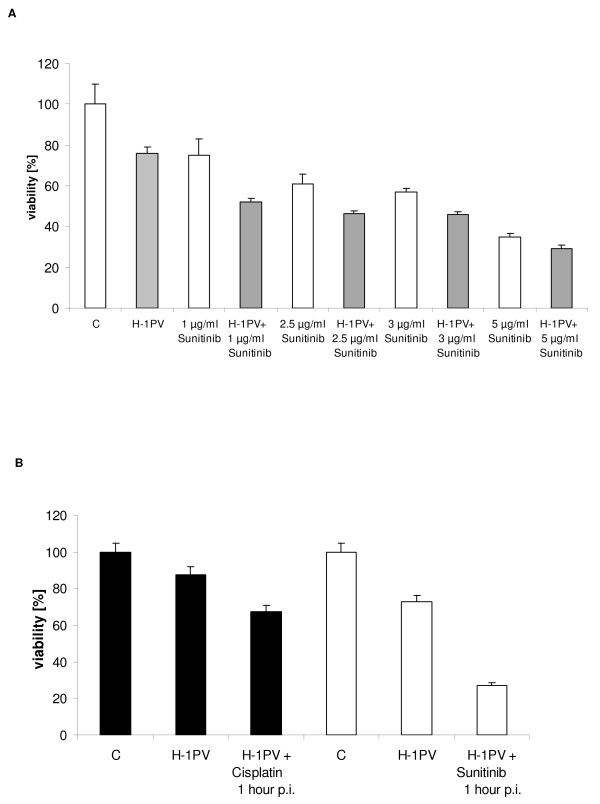
**SK29-Mel cell viability following H-1PV infection and/or treatment with sunitinib**. **A**: Cell viability of sunitinib treated and/or H-1PV-infected (MOI = 20 PFU/cell) SK29-Mel cells was measured by MTT assay after 48 h as in Figure 2. **B**: Cell viability of H-1PV (MOI = 20 PFU/cell), and/or chemotherapeutic or targeted agent-treated SK29-Mel cells 1 and 24 hours post infection was measured as in Figure 2.

We next quantified the effect of H-1PV infection in combination with sunitinib on cell viability of SK29-Mel cells. Sunitinib alone led to a decrease in SK29-Mel viability after 24 hours of treatment with the optimal concentration of 5 μg/ml. The combination of sunitinib with H-1PV led to a further reduction of SK29-Mel cell viability (Figure 3A) and was dependent on the time-point of exposure. Application 1 hour p.i. led to decreased cell viability of ~50% compared with H-1PV treatment alone (Figure 3B). In contrast, treatment 24 hours p.i. led to a 24% decrease in cell viability (data not shown). The combination of cisplatin with H-1PV also led to a reduction of cell viability, by 20% when administered at 1 hour p.i (Figure 3B) and by 23% administered 24 hour p.i. (data not shown), indicating no significant differences between administration of chemotherapeutic agents at 1 or 24 hour p.i. Thus, the combination of chemotherapeutic or targeted agents and H-1PV enhanced the reduction of SK29-Mel cell viability.

### Analysis of DC activity: production of inflammatory cyctokines and cross presentation

**Figure 4:**
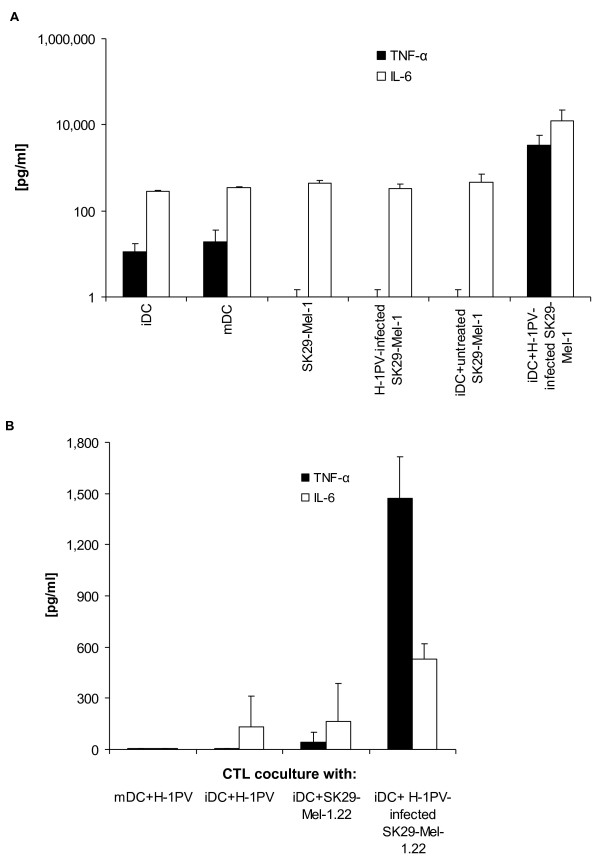
**Cytokine production in DC co-incubated with TCLs and from CTL co-cultured with DC incubated with TCLs**. **A**: TNF-α and IL-6 levels were determined in cell culture supernatants by ELISA. Culture was performed in 96 well plates. Immature DC were incubated for 1 day with untreated SK29-Mel-1 or H-1PV-infected SK29-Mel-1 cells 10 days p.i. (MOI = 20 PFU/cell). Controls included mature or immature DC alone and untreated SK29-Mel-1 cells or lysates in a ratio 1:10. **B**: CTL were co-cultured for 1 day in a 96-well plate either with lysate-incubated DC, DC alone, or lysates alone at a ratio of 1:10. Concentrations of TNF-α and IL-6 were determined in supernatants by ELISA.

We next compared cytokine release of DC co-cultured with different melanoma cell preparations (Figure 4A). Levels of TNF-α and IL-6 were increased by a factor of > 178 for TNF-α and a factor of > 36 for IL-6 when immature DC were co-cultured with H-1PV-induced SK29-Mel-1 cell lysates compared with control (mDC). Effects were also similar for the HLA-negative cell clone SK29-Mel-1.22 and MZ7-Mel cells (data not shown).

As immature DC can process HLA-negative tumors and present their TAAs in an HLA class I-restricted manner to tumor-specific CTL by cross presentation [32], we assessed whether phagocytosis of H-1PV-induced lysates mediates cross presentation of TAAs to CTLs. SK29-Mel-1.22 (HLA-A2^-^) were co-cultured with an A2^+^-restricted CTL to release cytokines on specific recognition of SK29-Mel TAAs. A dramatic increase in TNF-α levels following co-culture of CTL with DC incubated with H-1PV-induced SK29 Mel-1.22 lysates was observed (Figure 4B). The TNF-α level increased by a factor of > 32. High levels of IL-6 (increased by a factor of > 3) were also observed in CTL co-cultures with immature DC incubated with H-1PV-induced SK29-Mel-1.22 TCL (Figure 4B). CTL co-culture controls with immature DC and H-1PV, or immature DC and untreated SK29-Mel-1.22 led to the release of only small amounts of IL-6 and TNF-α, providing no evidence for cross-presentation. Mature DC and H-1PV alone did not increase the release of TNF-α or IL-6.

### Activation of DC by different SK29-Mel cell preparations

**Figure 5:**
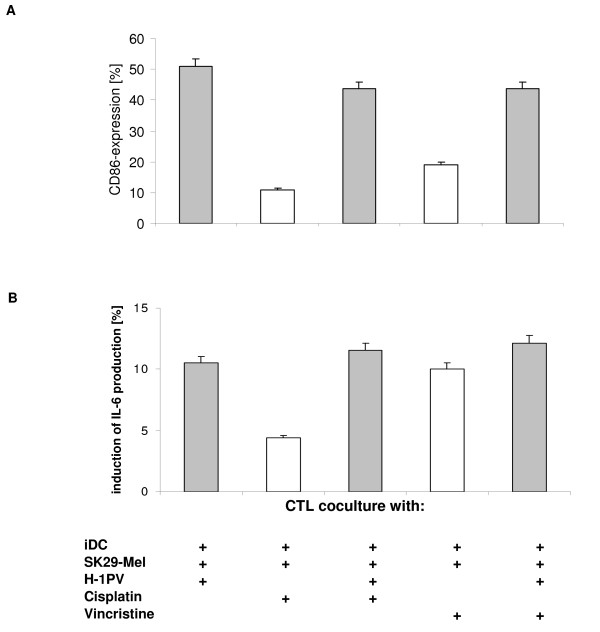
**DC maturation and cytokine production of CTL after incubation with H-1PV and chemotherapy-treated SK29-Mel cells**. **A**: Activation of DC by SK29-Mel cells treated with H-1PV (MOI = 20 PFU/cell) and/or chemotherapeutic agents. Immature DC were incubated for 2 days with different lysates as indicated. The maturation marker CD86 was measured via FACScan. **B**: CTL IL-6-production. CTL were co-cultured with TCL-incubated DC for 1 day in a 96-well plate. IL-6 levels were determined using ELISA.

To investigate the effects of different TCL preparations on DC maturation, CD86 was quantified using FACScan analysis. Immature DC were incubated for 2 days with differentially treated melanoma cells. Preparations from non-infected cells induced maturation in ~11% of DC (data not shown). However, TCL from H-1PV-infected melanoma cells led to 51% maturation of DC (Figure 5A). In contrast, cisplatin treatment alone of SK29-Mel cells was as effective in inducing DC maturation (11%) as untreated cells. Again, vincristinetreated SK29-Mel cells did not significantly enhance CD86 expression (19%). Immature DC incubated with SK29-Mel treated with a combination of H-1PV and vincristine or H-1PV and cisplatin enhanced CD86 expression compared with the agents alone, although this was not as great as that observed with TCL from H-1PV-infected SK29-Mel cells alone. These findings suggest that H-1PV infection of SK29-Mel cells compared with cisplatin and vincristine treatment led to a greater positive effect on DC maturation.

**Figure 6:**
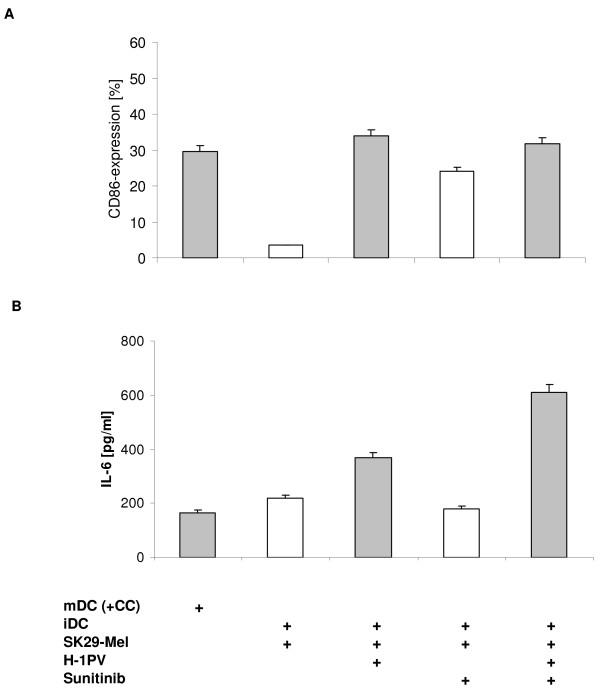
**DC maturation and cytokine production after incubation with H-1PV and sunitinib treated SK29-Mel cells**. **A**: Activation of DC by SK29-Mel cells treated with H-1PV (MOI = 20 PFU/cell) and/or sunitinib. Immature DC were incubated for 2 days with different lysates as indicated. Sunitinib 5 μg/ml was used. The maturation marker CD86 was measured via FACScan. **B**: CTL IL-6-production. CTL were co-cultured with TCL-incubated DC for 1 day in a 96-well plate. IL-6 levels were determined using ELISA.

In the same model, SK29-Mel cells treated with sunitinib alone led to a slight increase in CD86-expression in 24% of DC (Figure 6A). However again, CD86 expression was significantly enhanced in immature DC co-cultured with H-1PV-sunitinib-induced TCL (32%) compared with sunitinib alone. So DC maturation mediated using sunitinib-induced TCL can be clearly enhanced by additional infection of melanoma cells with H-1PV, providing a more effective immune response.

We lastly investigated the IL-6 production from CTL co-cultured with immature DC and both melanoma cells treated with chemotherapeutic agents and H-1PV. Stimulation of DC with H-1PV-induced TCL led to ~11% increase in IL-6 production, which was similar to that observed with H-1PV plus cisplatin or vincristine, but appeared higher than with cisplatin alone (Figure 5B). Of note, IL-6 levels were also increased after co-incubation of immature DC with sunitinib-treated SK29-Mel cells and H-1PV-induced lysates compared with H-1PV alone or sunitinib alone (Figure 6B). Very similar results were obtained with MZ7-Mel cells (data not shown).

## Discussion

Current novel anticancer strategies aim to enhance both apoptotic tumor cell death and immunologic tumor cell recognition. Therefore oncolytic viruses are of increasing clinical interest, [33,34] in particular, autonomous parvoviruses, which appear very promising for tumor targeting [8,10,26]. Since parvoviruses kill human cancer cells through different pathways to other anticancer therapies, we explored the impact and possible synergistic effects between H-1PV and chemotherapeutic agents or sunitinib, a typical molecule of the new multi-TK inhibitors [35]. Sunitinib has activity against the signalling pathways of vascular endothelial growth factor (VEGF)-receptors as well as RAF, platelet-derived growth factor receptor beta, fibroblast growth factor receptor and stem cell factor receptor (c-KIT) across a range of solid tumor types [36]. Preclinical and clinical studies linked the antitumoral effects of sunitinib with its inhibitory activity on these TKs [20].

We first confirmed that H-1PV-induced cell killing in MZ7-Mel cells correlates with NS1-expression which is consistent with other studies, and suggests that H-1PV has high potential to achieve tumor suppression [6,10,16,37]. Similarly, the combined treatment with H-1PV and gemcitabine lead to toxicity in cell lines regardless of used high doses of chemotherapeutic agent and impaired virus replication [38]. Additionally, combined treatment of the comparable adeno-associated virus AAV-2 with cisplatin showed enhanced apoptosis independent from cisplatin [33]. NS1 expression is essential for transgene expression driven by recombinant constructs. Previous studies have described how recombinant viruses containing *MCP3 *or *IL-2 *genes could stimulate the immune system leading to additional invasion of macrophages and NK cells in tumors [18]. Furthermore, the oncosuppressive capacity of CpG-armed H-1PV vectors was tested *in vivo *in a rat hepatoma cell metastatic model and the therapeutic vaccination effect of the vector was accompanied by a strong induction of cell mediated immune response [12].

We secondly combined antitumoral agents with oncolytic H-1PV. Here we investigated cisplatin and vincristine, commonly used as anticancer treatment either in combination or as monotherapy due to their different modes of action [34,39]. The antitumor properties of cisplatin are thought to be mediated via its ability to form DNA adducts, including intra-and interstrand DNA cross-links. Vincristine is a vinca-alkaloid, whose mode of action occurs primarily through blocking the polymerization of tubulin, which suppresses microtubule dynamics, halting mitosis and cell death [40]. The combination of H-1PV with cisplatin or with vincristine resulted in a reduced cell survival, which appeared additive at most doses studied. These cytotoxic effects may depend on the p53 status of the cell lines, as cytotoxicity of H-1PV has been shown to be more pronounced in p53-negative than in p53-positive cells [11]. SK29-Mel cells express the wildtype p53 gene [41] and a greater effect may be observed in p53-negative cell lines. On the other hand, Angelova et al showed that gemcitabine resistance could efficiently be overcome by H-1PV [38]. Again in a rabbit tumor model, the oncolytic pox virus and expression of hGM-CSF from the virus induced tumor-specific CTL and enhanced tumor-specific CTL and antitumoral efficacy [42]. Even more, the vaccinia virus (VV-CD) could demonstrate *in vivo *antitumor effects when administered with 5-Flurocytidin (5FC) even if 5-FC inhibited virus replication [43]. Furthermore in clinical trials with head and neck cancer patients the oncolytic virus ONYX-015 was combined with chemotherapy and clear benefits were observed in combination with cisplatin and 5-FU compared to patients treated with chemotherapy alone [44,45]. CPA, another alkylating agent like cisplatin, showed no effect on immune cell infiltrates, viral replication or viral transgene expression in experiments performed in non-obese diabetes/severe combined immunodeficient mice [46,47]. Rather combined immunosuppressive effects of CPA correlated with increased viral transgene expression [48] and replication [49,50] in a variety of tumor models. Moreover, adenvoiruses combined with cisplatin presented *in vitro *a significant increase of apoptosis of tumor cells but not normal cells in different tumor models [51,52].

We next showed that phagocytosis of H-1PV-infected MZ7-Mel-cells by DC was enhanced in concordance with our previous data from other virus-infected cells [6,8,53]. Thirdly, we analyzed DC maturation following incubation with H-1PV-induced TCL by measuring the expression of the maturation markers, CD80, CD83 and CD86. DC incubated with H-1PV-induced MZ7-Mel cell lysates resulted in a dramatic increase in the proportion of DC expressing maturation markers, supporting similar data with other melanoma models, and infection with the oncolytic reovirus [54]. However, other viruses have been reported to directly infect DC causing lysis and blocking important immune reactions [55].

We observed CTL activation following co-incubation with DC, which had phagocytosed H-1PV-infected SK29-Mel cells, which could be due to cross-presentation. Activation occurred even if H-1PV alone was unable to stimulate the DC and our data are supported by other studies [53,56]. The increased release of pro-inflammatory cytokines by DC indicates augmented CD4^+ ^and CD8^+ ^T-cell activation. *In vivo*, this may lead to a response against TAAs [6,10,32,54]. As H-1PV-induced melanoma lysates induced a much higher level of phagocytosis by DC than either irradiated (necrotic) or untreated cells, H-1PV may directly enhance immune stimulation, which has also been shown with other viruses [57]. Further, we observed enhanced release of IL-6. Finally, there was a dramatic increase of TNF-α and IL-6 following co-culture of CTL with DC and lysates from H-1PV-infected melanoma cells. This is consistent with data from other groups, where chemotherapeutic drugs like vincristine, paclitaxel or methotrexate enhanced DC maturation [58]. Pandha et al showed that treatment with cisplatin increased the cytotoxicity of oncolytic reovirus but effects on the immune system were negative. The cytokine production induced by reovirus was suppressed by cisplatin [59]. In our system, the comparable combination with H-1PV did not hinder the immune stimulatory effects.

We further investigated the use of sunitinib in combination with H-1PV in the melanoma cell line SK29-Mel. We demonstrated that sunitinib decreases SK29-Mel cell viability and that this is enhanced in combination with H-1PV. It has previously been shown than sunitinib does not affect DC phenotype;[60] however, Finke et al have reported impaired regulatory T-cell function with sunitinib [61]. Furthermore, pretreatment with sunitinib had no inhibitory effect on the ability of DC to stimulate allogenic lymphocyte proliferation [60]. The amount of viable immature DC were not affected by sunitinib treatment, and it did not show any inhibitory effects on maturation and function of DC, nor impair the induction of primary T-cell responses *in vivo*. Furthermore, it reduced the number of Tregs, which constitute a major immune suppressive burden in cancer immune therapy [60]. However, the effect of sunitinib on the function of human immune responses has not been evaluated in detail.

It could be postulated that combined oncolytic virotherapy can amplify the role of anti-angiogenic therapy by enhancing the effect of sunitinib and additional tumor cell lysis. Furthermore, DC maturation by cells treated with H-1PV, sunitinib and their combination could be stimulated. This effect could also be shown by the enhanced IL-6 production after the combined treatment with H-1PV and sunitinib.

## Conclusions

In conclusion, our experiments show that the combination of chemotherapeutic agents and the apathogenic oncolytic H-1PV may enhance the tumor targeting armamentarium and may preferentially attain immunologically based long-term remission of cancer [31,62]. Presentation of TAAs, CTL activation and anti-TAA responses should be presented as expectations for (i) greater immunomodulatory and killing effects of H-1PV compared with cisplatin and vincristine alone; and (ii) increased cell killing effects by the combined treatment with H-1PV and sunitinib. Furthermore, the use of oncolytic viruses and anti-angiogenic agents in the treatment of cancer could potentiate the particular effect. As the first *in vitro *proof of concept, these results indicate that the immunomodulatory properties of H-1PV are not disrupted by chemotherapeutic/targeted agents. Even more H-1PV oncolytic activity reinforced drug-induced tumor cell killing, suggesting the application of this combination in tumor therapy could afford new intriguing aspects in human cancer treatment.

## Abbreviations

H-1PV: parvovirus H-1; CTL: cytotoxic T-cell; DC: dendritic cell; TCL: tumor cell lysate; MOI: multiplicity of infection; TAA: tumor associated antigen; NK: natural killer cell; PBMC: peripheral blood mononuclear cells; TK: tyrosine kinase; MZ-EBV-B: EBV-transformed B-cell line; PFU: plaque-forming units; p.i.: post infection; ELISA: enzyme-linked immunosorbant assay; MTT: 2-(4,5-dimethyltriazol-2-yl)-2,5-diphenyl tetrazolium bromide; TNF-α: tumor necrosis factor-alpha; IL-6: interleukin-6; VEGF: vascular endothelial growth factor.

## Competing interests

The authors declare that they have no competing interests.

## Authors' contributions

The authors' contributions to this research work are reflected in the order shown, with the exception of MM who supervised the research and finalized the report. MS, SR, and FS carried out all of the experiments. MM and MS drafted the manuscript. MM and MS conceived of the study, and participated in its design and coordination. All authors read and approved the final manuscript.

## Pre-publication history

The pre-publication history for this paper can be accessed here:

http://www.biomedcentral.com/1471-2407/11/464/prepub
